# Epiphyte response to drought and experimental warming in an Andean cloud forest

**DOI:** 10.12688/f1000research.3-7.v2

**Published:** 2014-06-06

**Authors:** Joshua M. Rapp, Miles R. Silman

**Affiliations:** 1 Department of Biology, Wake Forest University, Winston Salem, NC, 27109, USA; 2 Current address: Department of Biology, Tufts University, Medford, MA, 02155, USA

## Abstract

The high diversity and abundance of vascular epiphytes in tropical montane cloud forest is associated with frequent cloud immersion, which is thought to protect plants from drought stress. Increasing temperature and rising cloud bases associated with climate change may increase epiphyte drought stress, leading to species and biomass loss. We tested the hypothesis that warmer and drier conditions associated with a lifting cloud base will lead to increased mortality and/or decreased recruitment of epiphyte ramets, altering species composition in epiphyte mats. By using a reciprocal transplant design, where epiphyte mats were transplanted across an altitudinal gradient of increasing cloud immersion, we differentiated between the effects of warmer and drier conditions from the more general prediction of niche theory that transplanting epiphytes in any direction away from their home elevation should result in reduced performance. Effects differed among species, but effects were generally stronger and more negative for epiphytes in mats transplanted down slope from the highest elevation, into warmer and drier conditions, than for epiphyte mats transplanted from other elevations. In contrast, epiphytes from lower elevations showed greater resistance to drought in all treatments. Epiphyte community composition changed with elevation, but over the timescale of the experiment there were no consistent changes in species composition. Our results suggest some epiphytes may show resistance to climate change depending on the environmental and evolutionary context. In particular, sites where high rainfall makes cloud immersion less important for epiphyte water-balance, or where occasional drought has previously selected for drought-resistant taxa, may be less adversely affected by predicted climate changes.

## Introduction

Tropical montane forests, often referred to as cloud forests, harbor high species diversity, provide water and protect water quality for numerous people in tropical countries, and are under particular threat from climate change ^1– 3^. Most cloud forest regions of the world, including the tropical Andes, are considered hotspots of biological diversity ^4^, and plant species endemism often reaches high levels within cloud forest ^5, 6^. Epiphytes, non-parasitic plants that depend on other plants for support and are not in contact with terrestrial soil, are key components of cloud forest biodiversity and play critical roles in the hydrological and nutrient cycling of montane ecosystems. Not only can vascular epiphytes make up 30 percent or more of plant species diversity in tropical montane forests ^7^, they also provide keystone resources for birds, insects, and other animals ^8– 11^. Through cloud stripping, epiphytes increase total moisture captured by forest canopies ^12– 14^, and are important in nutrient cycling ^15, 16^. Cloud immersion is important for many epiphyte species to maintain a positive water balance and avoid desiccation ^17^; this makes them sensitive to changes in moisture regimes. Because of their sensitivity to moisture levels, epiphytes are considered indicator species in cloud forests for changing water balance conditions ^18^, particularly those in the wet tropics ^19^.

On a typical tropical mountain, where temperature decreases with altitude, there is a gradient of increasing cloud incidence with altitude. Cloud formation is dependent on the vapor content of air and air temperature, both of which are predicted to change with global warming ^1^. Atmospheric moisture levels are much less easily predicted than temperature in climate models, but a multi-model ensemble of climate simulations showed a trend towards drying in many tropical regions ^20^. Climate model projections in the Andes include warmer temperatures and lower precipitation in the dry season ^21– 23^, which could place cloud forest plants under increased drought stress. Cloud base height is also predicted to rise, due to a combination of higher temperature and lower atmospheric moisture input by vegetation due to lowland deforestation and reduced transpiration because of increased atmospheric CO _2_^24– 26^. In Costa Rica, an increase in the elevation of cloud base has been demonstrated and has already lead to the extinction of cloud forest species ^27^, although this may have been associated with a severe El Niño event in 1986–87 rather than a long term drying trend ^28^.

The sensitivity of vascular epiphytes to changes in cloud incidence was demonstrated experimentally in Monteverde, Costa Rica, where vascular epiphytes transplanted below the cloud base had shorter lifespans and higher leaf mortality ^29^. It is not clear, however, whether this result can be generalized to continental cloud forests such as the eastern Andes. Cloud forests vary worldwide, with differences in cloud base height and the proportion of moisture received by the vegetation via cloud stripping versus rainfall ^30, 31^. Cloud forests near coastlines are heavily influenced by ocean conditions ^32^, while cloud formation on continental mountain ranges is dependent on moisture flux across continents driven by synoptic weather patterns. Deforested areas in Costa Rica have fewer clouds than adjacent forested areas ^33^, but simulations suggest that sea-surface temperature has a greater impact on lifting condensation level than deforestation ^34^. Cloud forests in continental mountains like the Andes are expected to be more sensitive to conditions of the adjoining lowland ecosystems, particularly deforestation ^25, 32^. In addition, complex topography in the eastern Andes and its interaction with prevailing winds leads to wet and dry areas within regions broadly considered cloud forest ^35^. Given the diversity of cloudiness and precipitation regimes that epiphytes as a group are exposed to, it is reasonable to expect that epiphytes may be adapted to local moisture regimes. For instance, epiphytes in lowland dry or seasonal forest have high desiccation tolerance ^36, 37^. Epiphytes in continental cloud forests like the Andes, which experience variable cloudiness regimes, may have more resistance to drought than those in locations with more stable cloud bases. Likewise, epiphytes growing at lower elevations, below the cloud base, may have greater drought tolerance than epiphytes growing above the cloud base.

Beginning in the 2005 austral winter (June and July), we conducted a year-long reciprocal transplant experiment across an elevational gradient in cloud formation in the eastern Andes of southern Peru to test the effect that cloud immersion has on the performance of vascular epiphytes. The reciprocal transplant design also allowed us to distinguish between the effects of moving mats away from their home elevation versus moving plants into lower moisture conditions. This is a key control, not often made climate change transplant studies [e.g. ^29, 38^], for if epiphytes are locally adapted, reduced performance is expected if moved in any direction from their bioclimatic optimum. The specific questions we addressed were: (1) Does demographic performance decline when epiphytes are moved farther from their home elevation? (2) Is this effect greater when transplanted down-slope into drier and warmer conditions as predicted by results from Costa Rica? ^29^

We focused on three aspects of demographic performance, ramet survival, recruitment, and change in population size. Ramet survival allows us to examine treatment effects on existing individuals, while ramet recruitment and population change gives insight into treatment effects on epiphyte populations. We expected that ramet survival and recruitment would decrease and population change become negative as mats were moved farther from their home elevation. We also hypothesized that the decrease in ramet survival and recruitment would be greater for mats moved down-slope than for mats moved upslope if moisture level is the dominant factor in determining epiphyte species distributions.

## Materials and methods

### Study area

The Kosñipata Valley (13°03′S, 71°33′W) lies along the eastern slope of the Andes in southern Peru. Elevations range from about 800 m to over 4000 m, and vegetation changes from pre-montane rainforest at the lowest elevations to tropical subalpine forest and puna (alpine grassland) at the highest elevations ^39^. The experiment was installed along a single forested ridge, with three transplant sites at 1500, 1650, and 1800 m. We chose these elevations because the large increase in vascular epiphyte and bryophyte biomass ^40^, and step changes in soil properties ^41^ and biomass carbon stocks ^42^ between 1500 and 2000 m elevation in the Kosñipata Valley are likely associated with higher cloud incidence and lower temperatures, as seen on other tropical mountains ^43, 44^. The bedrock underlying the ridge is Permian granite, and soils are classified as umbric Gleysols ^45^.

The Kosñipata Valley has a perhumid climate described in detail in Rapp and Silman ^46^. Temperature decreases linearly with altitude and annual rainfall is high across the gradient, with a distinct, but weak dry season (monthly rainfall > monthly potential evapotranspiration except during drought). Vapor pressure deficit (VPD) typically decreases with altitude across the east Andean slope above 1500 m for all months except June ^46^. While differences in VPD between 1500 m (lowest experimental elevation) and 1800 m (highest experimental elevation) are typically small, excursions to higher VPD (greater desiccation) are more severe at 1500 m ^46^. The dry season (May–August) is when cloud immersion is expected to be most important for cloud forest plants. In July, the driest month, high relative humidity associated with cloud immersion (>95%) is more common at 1840 m than at 1500 m at climate stations approximately 1 km from the study site, while vapor pressure deficits greater than 1.0 kPa are more common at the higher elevation ( Table 1). Vapor pressure deficits greater than 1.0 kPa are associated with moisture stress in cloud forest plants ^47, 48^. We took daily photographs at 16:00 local time from a fixed location at 1400 m facing up-valley towards the ridge were the experiment was installed during June and July 2005 and July 2006. We categorized cloud base height using these photographs as being less than 1500 m (below experimental elevations), 1500–1800 m (within experimental elevations), or greater than 1800 m (above the experimental elevations). This analysis confirmed that cloud base height did not differ between years (Χ ^2^ = 3.32, p = 0.19), and that cloud frequency increased with elevation (cloud base <1500 m in 9% of observations; 1500–1800 m in 30% of observations; >1800 in 61% of observations).

**Table 1.** July climate for weather stations within 1 kilometer of the transplant sites. Values represent the mean for July in 2007, 2008, and 2009, except for precipitation, which does not include data for 2007. RH95 is the proportion of time relative humidity was greater than 95%. VPD excursions is the number of days per month in which vapor pressure deficit (VPD) was 1.0 kPa or greater.

Elevation (m)	Temperature (°C)	Precipitation (mm/day)	Relative humidity (%)	RH95 (%)	VPD excursions (days/month)
1500	17.2	7.5	88.3	0.27	1.7
1840	16.1	4.6	88.4	0.32	0

### Data collection

In the 2005 austral winter (dry season: June and July), we selected five *Alzatea verticillata* Ruiz & Pav. trees at each of three elevations, 1500 meters, 1650 meters, and 1800 meters elevation. *Alzatea* was an appropriate choice for a host tree because: (1) it was common at all three elevations; (2) it attains large size and has strong wood suitable for supporting climbers working in the trees; and (3) its unique architecture resulted in many large horizontal branches that supported sizeable epiphyte mats. We accessed trees using roped arborist techniques ^49^. In each tree, we chose four sections of epiphyte mat that were at least 25 cm wide and 30–40 cm long. Within each of the 60 mats, we marked an area of 25 × 25 cm with wire, and marked all ramets of vascular epiphytes within it, identified them to morpho-species, and recorded the length of shoots and number of leaves of each ramet.

Most taxa, and all of the focal taxa (see below), were non-reproductive when surveyed. This, combined with the fact that the epiphytic flora of the Andes is relatively poorly known, made it impossible to identify all taxa to species. We therefore used morpho-species designations in our analysis. Taxonomic uncertainty therefore, could affect our results if individuals of multiple cryptic species were combined in the analysis. Nonvascular epiphytes were present, but not considered in this experiment.

Most mat dwelling epiphytes are clonal, with individual ramets connected by subsurface stems, but capable of surviving without connection to other ramets. It was difficult to determine individual genets without excavating the plants, so we identified and measured individual ramets rather than genetically distinct plants. For strap-leafed ferns in the genus *Elaphoglossum*, each ramet was a single leaf, while ramets for other taxa consisted of one or more stems with multiple leaves.

**Figure S1.**
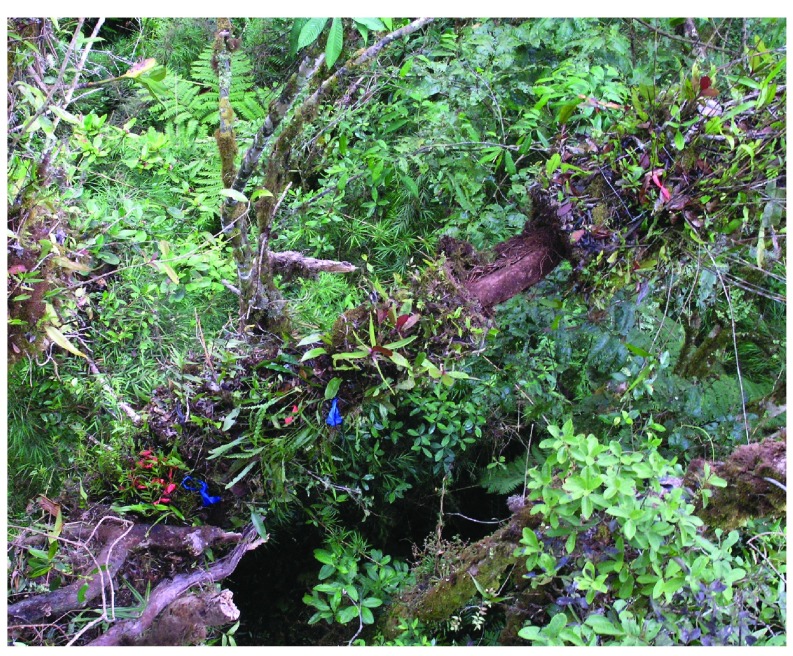
Study branch. Experimental mats in a tree at 1800 m were all on the same large horizontal branch. *From lower left to upper right*: two mats transplanted from trees at 1500 and 1650 m, an open space awaiting a transplanted mat from 1800 m, and a surveyed undisturbed control mat.

**Figure S4.**
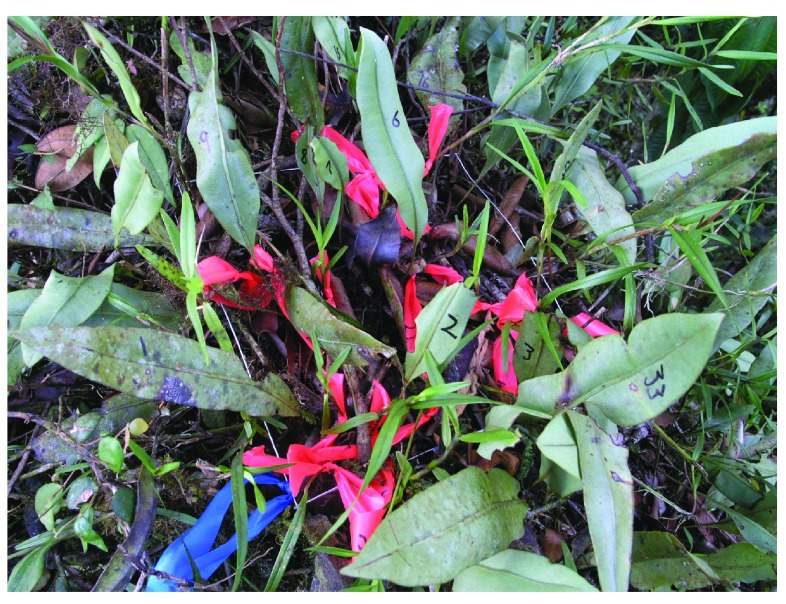
Control mat. A control epiphyte mat after being surveyed in 2005. This mat at 1800 m elevation included primarily *Elaphoglossum* and *Schaphyglottis*.

On each tree, one of the mats was left in place to serve as an undisturbed control. We cut each of the other three mats from the tree, lowered them to the ground, and then transplanted one each to a random tree at each of the three elevations. After all transplants were complete, each tree had one undisturbed mat, one mat that had been removed and then replaced at the same elevation, and one mat from each of the other two elevations. We tied each mat in place using wire, and then watered it with one liter of water to minimize any desiccation effect that handling may have had. Supplementary Figure 1– Supplementary Figure 4 illustrate the process of transplanting the epiphyte mats.

We left the mats undisturbed for one year, and resurveyed them the following year in June and July 2006 ( Dataset 1). We searched for all marked ramets and counted the new ramets of each morpho-species. We assumed ramets obviously more than a year old (28 out of 1400+ original ramets) had lost their tag if previously marked ramets of the same morpho-species were not found in the same mat. Eight ‘old’ ramets were still not accounted for; we assumed these were missed during the first census. Any other missing ramets were assumed to be dead.

Survey of epiphytes before and after transplantation across an altitudinal gradient of increasing cloud immersion along the eastern slope of the Andes, Peru. Full data set including all epiphyte ramets surveyed in 2005, before transplanting epiphyte mats, and in 2006, after transplantation. Columns are as follows: Mat.num, a unique number for each transplanted mat; From.elev, the elevation of the mat before transplantation; To.elev, the elevation of the mat after transplanting; From.tree, the tree the mat was in before transplantation; To.tree, the tree the mat was in after transplanting; Ramet.num, unique number for each ramet in a mat; mSpecies, morpho-species of the ramet; Guild, life-form of the ramet (fern, orchid, or shrub); Ramet.condition, condition of the ramet in 2006 (a: alive, d: dead, f: fresh, m: mature, n: new, s: senescent); treatment, undisturbed control or transplanted mat. Study location: Kosñipata Valley, Peru (13°03′S, 71°33′W). Data was collected between June-July in 2005 and 2006.

With these data, we defined three measures of population performance: 1) survival, 2) recruitment, and 3) population change. Survival was defined as:

            Survival = (N _2005_ – D _2006_)/N _2005_,

where N _2005_ was the number of ramets surveyed in a mat in each year, and D _2006_ was the number of ramets surveyed in 2005 that had died by 2006. Recruitment was defined as:

            Recruitment = n _2006_/N _2005_,

where n _2006_ was the number of new ramets surveyed in 2006, which were not present in 2005. Population change was defined as:

            Population change = (N _2006_ – N _2005_)/N _2005_.

### Focal species

We conducted analyses at the community level and for the most common morpho-species individually. The common morpho-species occurred in at least half (10) of all epiphyte mats transplanted from at least one elevation. These included four common morpho-species identified to genus, by which we will refer to them: a strap-leaf fern ( *Elaphoglossum* Schott ex J. Sm.), two orchid morpho-species ( *Maxillaria* Ruiz & Pav.; *Scaphyglottis* Poepp. & Endl), and an ericaceous shrub ( *Cavendishia* Lindl.). Collectively, these morpho-species accounted for 78% (1127/1452) of the ramets surveyed in the initial 2005 survey ( Table 2). *Elaphoglossum* was abundant across the gradient, *Maxillaria* and *Scaphyglottis* were most abundant at upper elevations, and *Cavendishia* was most common at the central elevation ( Table 2).

**Table 2.** Number of ramets in surveyed mats before transplanting. Numbers in parentheses indicate number of mats the ramets were found in. Bold indicates ramets used in single species analyses.

	Species					
Elevation (m)	*Elaphoglossum*	*Maxillaria*	*Cavendishia*	*Schaphyglottis*	All other species	*Total*
1500	**346 (19)**	16 (3)	11 (4)	0 (0)	181 (12)	554 (20)
1650	**163 (19)**	**118 (17)**	**46 (11)**	33 (8)	55 (10)	415 (20)
1800	**197 (19)**	**96 (14)**	20 (8)	**81 (10)**	89 (17)	483 (20)
*Total*	706 (57)	230 (34)	77 (23)	114 (18)	325 (39)	1452 (60)

### Statistical analysis

All analyses were performed using the mat as the experimental unit to account for within-mat correlations between ramets, i.e. to avoid pseudo replication. We fitted models to data that included all ramets irrespective of morpho-species to explore the overall community patterns of ramet survival, recruitment, and population change, and then modeled common morpho-species separately to look at individual morpho-species responses. We tested whether ramet recruitment was different than mortality in transplanted mats using a two-sided t-test. We then analyzed ramet survival, recruitment, and population change with respect to experimental manipulations using generalized linear mixed-effects models (GLMMs). Survival was modeled as a binomial distribution with a logit link function to account for the binary nature of the response (alive, dead). Recruitment (new ramets in 2006) and population change (total ramets in 2006) were modeled as a rate relative to the initial ramets per mat by using a Poisson distribution with a log link, and adding an offset of the log of the number of initial ramets in 2005. To account for the natural blocking by tree in our experimental design, models that included multiple source elevations and transplant elevations also included random effects for source tree and transplant tree. Models including only one source elevation included a random effect for source tree only. Likelihood ratio tests were used to assess the fixed effects, while Wald z-tests were used to evaluate differences between levels of fixed effects. We did not evaluate the significance of random effects because they were a required part of our experimental design. Finally, we confirmed that the residuals of the final model were not overdispersed ^50^ using code from Bolker *et al.* ^51^. All analyses were done in R [Version 2.15.2; 52]. In all analyses we considered an effect significant if the P-value was less than 0.05.

First, we tested for an effect of manipulating mats using data for undisturbed control mats and mats transplanted within elevation. Source elevation and treatment (transplant versus undisturbed) were modeled as fixed effects in this analysis. Then, we tested for effects of source and transplant elevation on the response variable, using data from just the transplanted mats. We took this two-tiered approach because a full model including all mats was unbalanced (e.g. there could not be a control mat that moved between elevations) and statistical models accounting for this would not converge computationally. For analysis of individual morpho-species, we used only source elevations for which the morpho-species was present in at least half (10) of the source mats from that elevation (see Table 2).

To investigate patterns in mat species composition we used Detrended Correspondance Analysis (DCA) because our compositional data collected across a directional gradient matched the assumptions of DCA. First, we investigated the change in composition versus elevation using the pre-transplantation composition of all mats. We then investigated compositional change due to experimental treatments by ordinating the composition of all mats during 2005 before transplantation, with the composition of mats in 2006, one year after transplantation. Permutation Multivariate Analysis of Variance using distance matrices [function adonis in the vegan R package; 53] was used to test for compositional changes with altitude and among years due to the transplantation.

## Results

### Transplant effect: transplants within elevation

**Figure 1.**
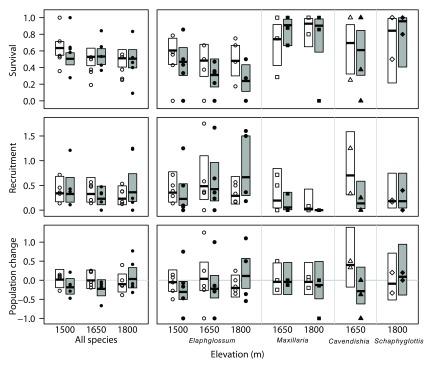
Survival, recruitment, and population change of ramets of all epiphyte species pooled and four abundant epiphyte species from a reciprocal transplant experiment within three elevations. Points show survival (top), recruitment (middle), and population change (bottom) of individual mats in 2006, each expressed as a proportion relative to the number of ramets present in 2005. Thick horizontal lines and boxes depict the modeled mean and 95% confidence intervals, respectively. White shading depicts controls, dark shading transplant.

First, we tested for an effect of transplantation independent of elevational distance moved by asking whether epiphytes in mats transplanted to the same elevation had different ramet survival, recruitment, and population change or turn-over than those in intact mats. Across all morpho-species, there was no significant effect of transplant, elevation, or their interaction on survival, recruitment, or population change of epiphyte ramets ( Table 3, Figure 1). However, individual morpho-species were affected by transplantation. For *Elaphoglossum*, survival was lower in mats transplanted to another site at the same elevation than in undisturbed controls, but there was no effect of elevation on survival or any interaction between elevation and transplantation ( Table 4, Figure 1). There was an interaction between elevation and transplantation for recruitment and population change in *Elaphoglossum*, however ( Table 4); both were lower for transplanted mats at 1500 m and 1650 m, but higher for mats transplanted at 1800 m ( Figure 1). For *Maxillaria*, recruitment was lower in transplanted mats, but not affected by elevation, and neither survival nor population change was affected by either transplanting or elevation ( Table 4, Figure 1). For *Cavendishia*, recruitment and population change were lower for transplanted mats ( Figure 1), but only significantly so for recruitment; survival was unaffected by transplantation ( Table 4). Transplanting did not affect survival, recruitment, or population change in *Scaphyglottis* ( Table 4, Figure 1).

**Table 3.** Results of generalized linear mixed-effects models (GLMMs) for all species in mats transplanted within elevations. SE: standard error.

	Estimate	SE	Statistic	P
*(a) Survival*				
Intercept	0.16	0.08		
Treatment			Χ ^2^_(1)_ = 2.15	0.142
Transplant vs. Control	-0.23	0.15	z = -1.47	0.143
Elevation			Χ ^2^_(2)_ = 1.20	0.548
1650 m vs. 1500 m	-0.11	0.18	z = -0.63	0.530
1800 m vs. 1500 m	-0.20	0.19	z = -1.07	0.287
1800 m vs. 1650 m	-0.09	0.21	z = -0.42	0.675
Treatment × elevation			Χ ^2^_(2)_ = 2.86	0.239
(levels not shown)				
Source tree [R]	0.00			
Transplant tree [R]	0.00			
*(b) Recruitment*				
Intercept	-1.20	0.20		
Treatment			Χ ^2^_(1)_ = 0.00	0.983
Transplant vs. Control	0.00	0.14	z = -0.02	0.982
Elevation			Χ ^2^_(2)_ = 0.13	0.938
1650 m vs. 1500 m	-0.17	0.49	z = -0.35	0.729
1800 m vs. 1500 m	-0.13	0.50	z = -0.25	0.801
1800 m vs. 1650 m	0.05	0.50	z = 0.09	0.927
Treatment × elevation			Χ ^2^_(2)_ = 4.53	0.104
(levels not shown)				
Source tree [R]	0.47			
Transplant tree [R]	0.56			
*(c) Population change*				
Intercept	-0.08	0.06		
Treatment			Χ ^2^_(1)_ = 2.18	0.140
Transplant vs. Control	-0.12	0.08	z = -1.48	0.138
Elevation			Χ ^2^_(2)_ = 0.32	0.852
1650 m vs. 1500 m	-0.03	0.14	z = -0.20	0.842
1800 m vs. 1500 m	0.05	0.14	z = 0.37	0.710
1800 m vs. 1650 m	0.08	0.14	z = 0.56	0.575
Treatment × elevation			Χ ^2^_(2)_ = 4.45	0.108
(levels not shown)				
Source tree [R]	0.13			
Transplant tree [R]	0.10			

[R] indicates random effect

**Table 4.** Results of generalized linear mixed-effects models (GLMMs) for ramet survival, recruitment, and population change of the four most abundant species in control mats and mats transplanted within elevations. SE: standard error.

	Survival				Recruitment				Population change			
	Estimate	SE	Statistic	P	Estimate	SE	Statistic	P	Estimate	SE	Statistic	P
*(a) Elaphoglossum*												
Intercept	0.1	0.23			-1.04	0.39			-0.05	0.15		
Treatment			Χ ^2^_(1)_ = 9.03	0.003			Χ ^2^_(1)_ = 0.14	0.713			Χ ^2^_(1)_ = 0.86	0.353
Transplant vs. Control	-0.77	0.24	z = -3.17	0.0015	-0.44	0.34	z = -1.32	0.187	-0.31	0.18	z = -1.70	0.089
Elevation			Χ ^2^_(2)_ = 2.48	0.29			Χ ^2^_(2)_ = 0.61	0.738			Χ ^2^_(2)_ = 0.24	0.886
1650 m vs. 1500 m	-0.57	0.45	z = -1.28	0.202	0.31	0.57	z = 0.55	0.581	0.09	0.23	z = 0.39	0.699
1800 m vs. 1500 m	-0.73	0.44	z = -1.67	0.094	-0.2	0.58	z = -0.35	0.727	-0.17	0.23	z = -0.76	0.447
1800 m vs. 1650 m	-0.16	0.47	z = -0.34	0.732	-0.51	0.59	z = -0.87	0.384	-0.26	0.25	z = -1.05	0.293
Treatment × elevation			Χ ^2^_(2)_ = 0.74	0.689			Χ ^2^_(2)_ = 7.96	0.019			Χ ^2^_(2)_ = 5.98	0.05
(levels not shown)												
Source tree [R]	0.32				0.52				0.14			
Transplant tree [R]	0.52				0.52				0.13			
*(b) Maxillaria*												
Intercept	2.35	0.75			-2.72	0.87			-0.06	0.11		
Treatment			Χ ^2^_(1)_ = 1.18	0.277			Χ ^2^_(1)_ = 3.48	0.062			Χ ^2^_(1)_ = 0.03	0.86
Transplant vs. Control	0.8	0.71	z = 1.13	0.26	-1.32	0.84	z = -1.58	0.115	-0.04	0.22	z = -0.16	0.87
Elevation			Χ ^2^_(1)_ = 0.25	0.617			Χ ^2^_(1)_ = 1.91	0.167			Χ ^2^_(1)_ = 0.03	0.86
1800 m vs. 1650 m	0.63	1.36	z = 0.46	0.642	-2.18	1.55	z = -1.41	0.16	-0.04	0.22	z = -0.16	0.87
Treatment × elevation			Χ ^2^_(1)_ = 1.17	0.279			Χ ^2^_(1)_ = 0.33	0.565			Χ ^2^_(1)_ = 0.04	0.848
(levels not shown)												
Source tree [R]	0.65				1.71				0			
Transplant tree [R]	1.57				0				0			
*(c) Cavendishia*												
Intercept	0.65	0.56			-0.35	0.41			0.34	0.27		
Treatment			Χ ^2^_(1)_ = 0.10	0.75			Χ ^2^_(1)_ = 4.86	0.028			Χ ^2^_(1)_ = 2.69	0.101
Transplant vs. Control	-0.38	0.97	z = -0.39	0.699	-1.63	0.83	z = -1.98	0.048	-0.67	0.41	z = -1.63	0.104
Source tree [R]	0.68				0.2				0			
*(d) Scaphyglottis*												
Intercept	2.23	1.41			-1.71	0.5			0	0.21		
Treatment			Χ ^2^_(1)_ = 1.17	0.279			Χ ^2^_(1)_ = 0	1			Χ ^2^_(1)_ = 0.18	0.67
Transplant vs. Control	1.4	1.37	z = 1.03	0.304	0	1	z = 0	1	0.18	0.43	z = 0.43	0.67
Source tree [R]	1.66				0				0			

[R] indicates random effect

### Transplants across elevation

**Figure 2.**
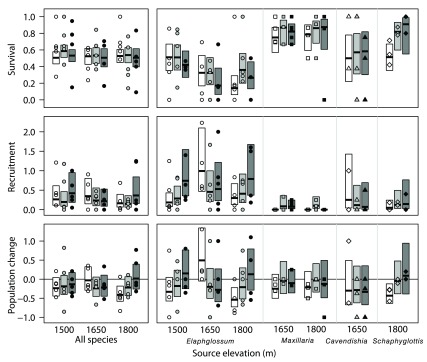
Survival, recruitment, and population change of epiphyte ramets from a reciprocal transplant experiment across three elevations. Points show survival (top), recruitment (middle), and population change (bottom) of individual mats in 2006, each expressed as a proportion relative to the number of ramets present in 2005. Thick horizontal lines and boxes depict the modeled mean and 95% confidence intervals, respectively. White shading depicts 1500 m elevation, light grey 1650 m elevation and dark grey 1800 m elevation.

Across all morpho-species, there were no significant effects on survival of any of the treatments for mats transplanted across elevations ( Table 5 and Figure 2). For recruitment and population change, there was a significant interaction between source and transplant elevation ( Table 5), with both positively associated with elevation for mats transplanted from 1500 and 1800 m, but negatively associated with altitude for mats from 1650 m ( Figure 2). Overall for transplanted mats, more ramets died than were recruited (mean change number of ramets per mat between years = -1.38; two-sided t-test, *P* = 0.01).

**Table 5.** Results of generalized linear mixed-effects models (GLMMs) for all species in mats transplanted between elevations. SE: standard error.

	Estimate	SE	Statistic	P
*(a) Survival*				
Intercept	0.13	0.06		
Source elevation			Χ ^2^_(2)_ = 0.31	0.858
1650 m vs. 1500 m	-0.07	0.15	z = -0.50	0.619
1800 m vs. 1500 m	-0.06	0.14	z = -0.41	0.683
1800 m vs. 1650 m	0.02	0.16	z = 0.11	0.916
Transplant elevation			Χ ^2^_(2)_ = 1.64	0.440
1650 m vs. 1500 m	0.16	0.14	z = 1.17	0.241
1800 m vs. 1500 m	0.01	0.14	z = 0.07	0.947
1800 m vs. 1650 m	-0.15	0.15	z = -1.03	0.301
Source elevation × transplant elevation			Χ ^2^_(4)_ = 1.09	0.895
(levels not shown)				
Source tree [R]	0.00			
Transplant tree [R]	0.00			
*(b) Recruitment*				
Intercept	-1.33	0.42		
Source elevation			Χ ^2^_(2)_ = 0.41	0.816
1650 m vs. 1500 m	0.27	0.48	z = 0.57	0.570
1800 m vs. 1500 m	-0.48	0.51	z = -0.94	0.345
1800 m vs. 1650 m	-0.46	0.51	z = -0.89	0.373
Transplant elevation			Χ ^2^_(2)_ = 2.37	0.305
1650 m vs. 1500 m	-0.29	0.42	z = -0.69	0.492
1800 m vs. 1500 m	0.47	0.40	z = 1.16	0.244
1800 m vs. 1650 m	-0.09	0.46	z = -0.20	0.845
Source elevation × transplant elevation			Χ ^2^_(4)_ = 13.31	0.010
(levels not shown)				
Source tree [R]	0.66			
Transplant tree [R]	0.53			
*(c) Population change*				
Intercept	-0.27	0.16		
Source elevation			Χ ^2^_(2)_ = 0.30	0.860
1650 m vs. 1500 m	0.24	0.18	z = 1.36	0.174
1800 m vs. 1500 m	-0.26	0.19	z = -1.41	0.157
1800 m vs. 1650 m	0.03	0.18	z = 0.15	0.885
Transplant elevation			Χ ^2^_(2)_ = 1.03	0.597
1650 m vs. 1500 m	0.06	0.19	z = 0.33	0.742
1800 m vs. 1500 m	0.13	0.19	z = 0.67	0.506
1800 m vs. 1650 m	0.03	0.21	z = 0.13	0.898
Source elevation × transplant elevation			Χ ^2^_(4)_ = 13.57	0.009
(levels not shown)				
Source tree [R]	0.17			
Transplant tree [R]	0.21			

[R] indicates random effect

*Elaphoglossum* ramets in mats originating at 1500 m had consistently and significantly higher survival than those originating at 1650 m or 1800 m, but there was no effect of transplant elevation on survival ( Table 6, Figure 2). For both recruitment and population change, however, there was a significant interaction between source elevation and transplant elevation, with both recruitment and population change declining in mats transplanted at lower elevations for mats originating at 1500 m and 1800 m, but for mats originating at 1650 m recruitment was greater and population change more positive for mats transplanted to 1500 m than for mats transplanted to higher elevation ( Table 6, Figure 2).

**Table 6.** Results of generalized linear mixed-effects models (GLMMs) for ramet survival of the four most abundant species in mats transplanted between elevations. SE: standard error.

	Survival				Recruitment				Population change			
	Estimate	SE	Statistic	P	Estimate	SE	Statistic	P	Estimate	SE	Statistic	P
*(a) Elaphoglossum*												
Intercept	-0.97	0.31			-1.68	0.42			-0.4	0.22		
Source elevation			Χ ^2^_(2)_ = 8.28	0.02			Χ ^2^_(2)_ = 1.80	0.407			Χ ^2^_(2)_ = 1.21	0.547
1650 m vs. 1500 m	-0.92	0.36	z = -2.57	0.01	1.68	0.43	z = 3.94	<0.001	0.8	0.24	z = 3.32	0.001
1800 m vs. 1500 m	-1.07	0.35	z = -3.02	0	0.47	0.45	z = 1.05	0.292	-0.37	0.28	z = -1.34	0.181
1800 m vs. 1650 m	-0.15	0.39	z = -0.38	0.7	-0.11	0.43	z = -0.26	0.795	-0.01	0.28	z = -0.03	0.974
Transplant elevation			Χ ^2^_(2)_ = 1.34	0.51			Χ ^2^_(2)_ = 2.74	0.254			Χ ^2^_(2)_ = 1.87	0.393
1650 m vs. 1500 m	0.31	0.37	z = 0.85	0.39	0.43	0.5	z = 0.86	0.389	0.21	0.28	z = 0.73	0.465
1800 m vs. 1500 m	-0.13	0.38	z = -0.34	0.74	1.38	0.49	z = 2.83	0.005	0.54	0.28	z = 1.92	0.055
1800 m vs. 1650 m	-0.44	0.38	z = -1.17	0.24	0.15	0.52	z = 0.29	0.774	-0.1	0.35	z = -0.29	0.772
Source elevation × transplant elevation			Χ ^2^_(4)_ = 5.86	0.21			Χ ^2^_(4)_ = 24.42	<0.001			Χ ^2^_(4)_ = 24.79	<0.001
(levels not shown)												
Source tree [R]	0.37				0.4				0.19			
Transplant tree [R]	0.49				0.57				0.32			
*(b) Maxillaria*												
Intercept	1.52	0.2			-21.33	5515.88			-0.14	0.08		
Source elevation			Χ ^2^_(1)_ = 0.20	0.658			Χ ^2^_(1)_ = 0.23	0.632			Χ ^2^_(1)_ = 0.00	0.946
1800 m vs. 1650 m	0.19	0.41	z = 0.45	0.651	-0.38	0.83	z = -0.46	0.645	0.02	0.17	z = 0.10	0.918
Transplant elevation			Χ ^2^_(2)_ = 1.95	0.377			Χ ^2^_(2)_ = 5.97	0.051			Χ ^2^_(2)_ = 1.27	0.529
1650 m vs. 1500 m	0.67	0.51	z = 1.32	0.187	18.86	5515.88	z = 0.00	0.997	0.23	0.2	z = 1.11	0.268
1800 m vs. 1500 m	0.44	0.48	z = 0.93	0.353	18.39	5515.88	z = 0.00	0.99	0.15	0.21	z = 0.73	0.464
1800 m vs. 1650 m	-0.23	0.55	z = -0.42	0.674	-0.47	0.83	z = -0.56	0.576	-0.08	0.2	z = -0.38	0.704
Source elevation × transplant elevation			Χ ^2^_(2)_ = 0.17	0.917			Χ ^2^_(2)_ = 1.78	0.412			Χ ^2^_(2)_ = 0.03	0.986
(levels not shown)												
Source tree [R]	0				0				0			
Transplant tree [R]	0				0.49				0			
*(c) Cavendishia*												
Intercept	0.22	0.34			-1.97	0.45			-0.36	0.2		
Transplant elevation			Χ ^2^_(2)_ = 0.18	0.916			Χ ^2^_(2)_ = 0.77	0.681			Χ ^2^_(2)_ = 0.02	0.989
1650 m vs. 1500 m	0.29	0.83	z = 0.35	0.729	-0.77	1.14	z = -0.67	0.502	0.02	0.49	z = 0.04	0.967
1800 m vs. 1500 m	0.34	0.86	z = 0.39	0.696	-1.26	1.4	z = -0.90	0.368	-0.05	0.52	z = -0.09	0.925
1800 m vs. 1650 m	0.05	0.8	z = 0.06	0.951	-0.49	1.29	z = -0.38	0.702	-0.07	0.47	z = -0.15	0.884
Source tree [R]	0				0				0			
*(d) Scaphyglottis*												
Intercept	0.05	0.33			-2.44	0.54			-0.26	0.14		
Transplant elevation			Χ ^2^_(2)_ = 9.81	0.007			Χ ^2^_(2)_ = 1.64	0.44			Χ ^2^_(2)_ = 4.37	0.112
1650 m vs. 1500 m	1.45	0.64	z = 2.25	0.024	0.95	0.95	z = 1.01	0.314	0.52	0.31	z = 1.69	0.092
1800 m vs. 1500 m	2.25	1.1	z = 2.05	0.041	1.1	1.03	z = 1.07	0.285	0.65	0.36	z = 1.81	0.071
1800 m vs. 1650 m	0.8	1.19	z = 0.67	0.501	0.15	0.96	z = 0.15	0.877	0.13	0.36	z = 0.37	0.712
Source tree [R]	0				0.63				0			

[R] indicates random effect

For *Maxillaria*, the only significant effect for transplanted mats was that for transplant elevation on recruitment ( Table 6); recruitment was low in all transplanted mats, but there was zero recruitment in mats transplanted to 1500 m ( Figure 2). There were no significant effects of source elevation or transplant elevation on survival or population change ( Table 6), but survival was lower and population change more negative for ramets transplanted to 1500 m ( Figure 2).

All three measures of performance were unaffected by transplant elevation in *Cavendishia* ( Table 6, Figure 2). For *Scaphyglottis*, survival, recruitment, and population change were all progressively lower in mats transplanted to lower elevations ( Figure 2), but the difference was significant only for survival ( Table 6).

### Community composition

**Figure 3.**
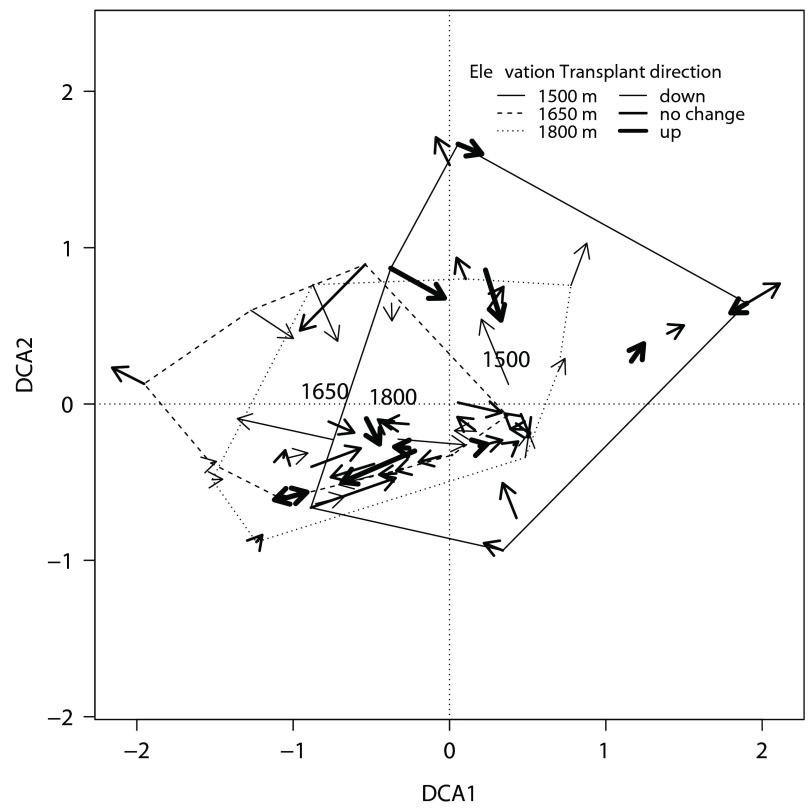
First two axis of a Detrended Correspondance Analysis (DCA) on the species composition of epiphyte mats both before and after mats were transplanted. Arrows connect the compositions of individual mats before and a year after transplantation. Line width depicts direction of transplanting. Hulls are drawn around the 2005 composition of mats that originated at the same elevation, and labels are placed at the hull centroid.

Prior to transplanting mats, the epiphyte community composition showed significant differences across the elevational gradient, although relatively little of the variation could be explained by elevation ( Table 7); most of the compositional separation was between mats at 1500 m and the other two elevations ( Figure 3). Morpho-species richness increased with elevation (Poisson regression, Z = 2.446, *P =* 0.0144), while the number of ramets per mat declined (Poisson regression, Z = -2.281, *P* = 0.0225; Table 8). Comparison of pre- and post-treatment species compositions in mats revealed no directional shift in community composition due to transplantation ( Table 7). A few mats did show large changes ( Figure 3), likely because of large changes in abundance in *Elaphoglossum*, either through high ramet mortality or recruitment ( Figure 2).

**Table 7.** ANOVA table from permutational multivariate Analysis of Variance to test differences in composition between mats at different elevations across years and treatments.

Source of variation	df	SS	MS	F	R ^2^
Elevation	1	0.75	0.75	3.38	0.0283**
Treatment	1	0.25	0.25	1.14	0.0095
Year	1	0.071	0.07	0.32	0.0027
Elevation × Treatment	1	0.17	0.17	0.74	0.0062
Elevation × Year	1	0.31	0.31	1.40	0.0117
Treatment × Year	1	0.04	0.04	0.20	0.0016
Elevation × Treatment × Year	1	0.10	0.10	0.44	0.0037
Residuals	112	24.92	0.22		0.9363
Total	119	26.61			1

Significance levels: **p < 0.01

**Table 8.** Species richness or morpho-species and ramet density of mats surveyed in 2005. Per mat values are means with standard error in parentheses.

Elevation (m)	Total species	Species per mat	Ramets per mat
1500	13	2.35 (1.04)	27.70 (11.20)
1650	14	3.65 (1.14)	20.75 (6.82)
1800	16	3.75 (1.16)	24.15 (11.81)

## Discussion

Vascular epiphytes transplanted down slope from our highest elevation had lower ramet recruitment and the number of ramets declined ( Table 5, Figure 2) when transplanted to the lowest elevation, suggesting warmer temperatures and lower cloud immersion will cause community-level changes for species currently above the cloud base. This result corroborates previous work in another tropical montane site, which found fewer leaves and shorter life-spans for vascular epiphytes moved down slope ^29^. However, reciprocal transplants between all elevations revealed unexpected dynamics, with demographic rates differing in their response and morpho-species responding individualistically to the treatments ( Figure 2). In general, survival was less sensitive than recruitment; for all ramets combined there was a significant interaction between source and transplant elevation for ramet recruitment and population change, but not for survival ( Table 5). Morpho-species also differed in the strength of their response to transplantation across elevation. *Cavendishia*, a small woody shrub showed the least response, while *Elaphoglossum*, a strap-leafed fern in which individual leaves were the measurement unit, was most responsive to treatments; there were significant effects for both ramet recruitment and population change ( Table 6). The two orchid morpho-species were intermediate, with *Scaphyglottis* responding more strongly (significant effect for survival, Table 6) than *Maxillaria* which has stouter stems.

Given the relatively short 1-year duration of the experiment, the relatively modest effects observed should perhaps be expected. Stronger effects would be expected for an experiment carried out over multiple years, since plants often react to stressful conditions through physiological responses such as closing stomata, which lowers carbon acquisition ^54– 57^. While this could eventually lead to mortality, plants are likely to first lower investment in growth and reproduction ^57^. In this context, it is not surprising that recruitment was more responsive that survival. It is also possible that functional differences in ramet construction may account for the differences in response among morpho-species to the elevational transplants, although our experiment was not set up to test this hypothesis directly. More species in each functional type would be needed to rigorously test this, as well as physiological measurements to demonstrate functional differences among species.

In general, it appears that epiphytes responded to water stress and/or higher temperatures but we also found evidence for local adaptation. The response to transplanting was strongest in those transplanted from the highest elevation, which is coolest and has the highest degree of cloud immersion. Epiphytes from lower elevations only benefitted slightly from increased water availability and cooler temperatures, possibly indicating they are better adapted to withstand heat and drought stress. Epiphytes from the middle elevations, where temperatures and cloud immersion are intermediate, responded in more idiosyncratic ways to transplantation. Finally, while composition changed across the elevational gradient, there was no significant directional shift in composition due to any of the transplant treatments ( Figure 3, Table 7). The relative resistance of epiphytes to expected transplant-induced moisture stress found in this study could be due to two competing factors, described below in more detail: (1) rainfall compensation in this pluvial system, where high rainfall is sufficient to maintain epiphyte water-balance below cloud base; and/or (2) higher epiphyte drought tolerance from a history of variable rainfall and occasional drought in these continental mountains. While these factors act in opposite directions, both are plausible mechanisms for epiphyte resilience to decreased cloud immersion. It is even possible that they work in concert, with rainfall compensation maintaining epiphyte water-balance in most years, while occasional drought provides a selective pressure for drought tolerance. We describe each of these mechanisms in detail below.

### Rainfall compensation

While our results for epiphytes transplanted from the highest elevation are consistent with the hypothesized altitudinal gradient in moisture stress, this gradient had less of an effect on epiphyte performance than the one in Monteverde, Costa Rica ^29^. The relative importance of cloud immersion for the distribution of epiphytes in this system may account for the difference. A consistent cloud base is a significant feature of many tropical montane forests ^2, 58^, and regular low cloud is assumed to maintain the diversity and abundance of cloud forest epiphytes, and control many of the unique structural and functional features of cloud forests ^43, 44^. Indeed, we chose the elevations for this experiment because of a suite of changes in ecosystem structure and function that occur at these elevations, including a step-change in bryophyte and vascular epiphyte biomass ^40^, tree height, above ground biomass, and forest productivity declining ^42^, and soil organic matter increasing ^41^ above 1500 m. Tree diversity also begins to decline above 1500 m in the study region ^59, 60^ mimicking the general pattern in the Andes ^61, 62^. These clear changes in forest structure, diversity and productivity contrast with smoother changes in climate. Mean temperature, precipitation, and VPD, a measure of moisture stress on plants, all decrease linearly with elevation above 1000 m ^46^.

High rainfall in this part of the Andes may mean that epiphytes here are less dependent on cloud immersion to maintain their water balance than their counterparts in other cloud forests. Even in 2005 under drought conditions, total precipitation for the year was 3273 mm. In this pluvial system, cloud base may be less important in determining epiphyte distributions than in other systems. It is noteworthy that the Nadkarni and Solano ^29^ experiment was carried out on the leeward Pacific slope of Monteverde, which is drier than the Caribbean slope ^63, 64^. Mean annual precipitation on the Pacific slope is 2155 mm at 1480 m in the cloud forest ^65^, and declines at lower elevations ^64^, and there is a 5–6 month dry season where much of the hydrologic balance is maintained by cloud immersion ^65^. This steep moisture gradient between cloud forest and lower elevations probably leads to a greater dependence of epiphytes on cloud immersion. If this previous study had been carried out on the Caribbean slope, where precipitation is higher at lower elevation ^64^, the results may have been similar to our study. On leeward slopes rainfall compensation may occur, in which epiphyte survival is enhanced by high rainfall even when there is less frequent cloud immersion.

### Drought tolerance

**Figure 4.**
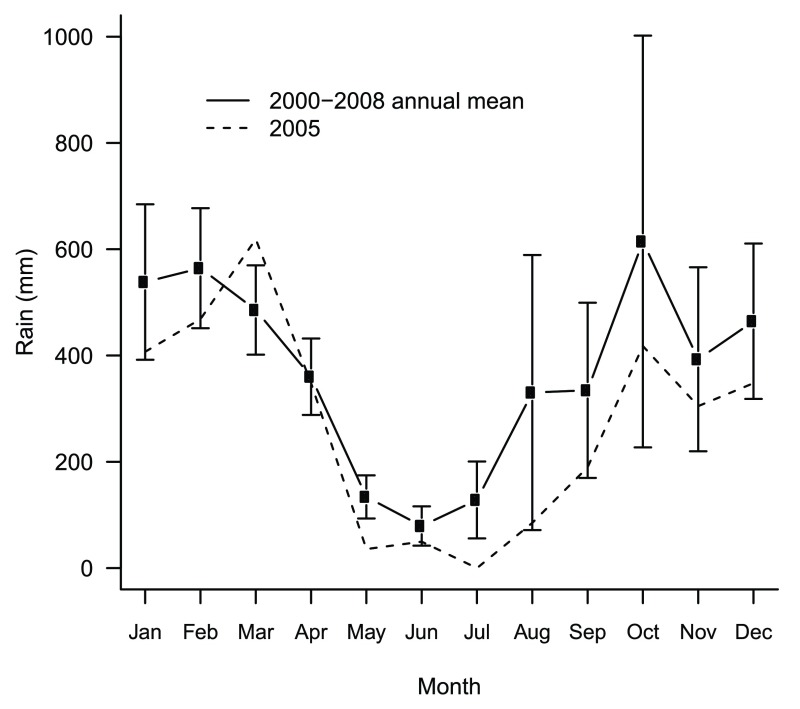
Monthly precipitation (mm) recorded at the Rocotal meteorological station at 2010 m maintained by SENAMHI in the Kosñipata Valley. Monthly means with 95% confidence intervals are shown for 2000–2008 exclusive of 2005, and compared with 2005 monthly totals.

Even though high rainfall may maintain epiphytes under normal conditions in the eastern Andes, droughts do occur, and epiphytes may be adapted to infrequent drought, especially at the lower fringe of the cloud forest. Drought in the Amazon basin during 2005 ^66, 67^ resulted in lower precipitation in the cloud forest. Although microclimate data were not available at the experimental elevations during the study, rainfall at the Peruvian SENAMHI meterological station at Rocotal (13°06′41″S, 71°34′14″, approximately 7 km from the transplant site at 2010 m elevation) for May–August in 2005 was the lowest for any year measured (mean May–August precipitation for 2000–2008: 601 mm; 2005: 175 mm; Figure 4). There was no recorded rainfall in July 2005, the only month during the nine-year measurement period with no recorded precipitation (mean July precipitation: 112 mm). In addition, actual cloud water interception based on fog collectors in place during the experiment did not show a gradient of increasing moisture with elevations during the 2005 dry season (four week total weight of water collected: 1500 m, 1109 g m ^-2^; 1750 m, 35 g m ^-2^; 1900 m, 72 g m ^-2^). All elevations were very dry, and desiccation was evident in bryophytes and non-succulent vascular epiphytes in the study area. However, ramet mortality in undisturbed control mats was not significantly greater than recruitment at any elevation ( Figure 1). In addition, mat species composition did not change directionally between years ( Figure 3). Thus, undisturbed epiphytes between 1500 and 1800 m in this Andean cloud forest appeared resistant to drought over the one-year time scale of our experiment.

This resistance to drought may be related to the normally variable and seasonal rainfall at the study site. Annual rainfall totals ranged between 3 and 6 m per year in a five year period not including the 2005 Amazonian drought. Precipitation was lowest in June and July ^46^, when temporary drought is possible, though for no month did potential evapotranspiration exceed precipitation at these elevations in most years. However, prolonged (days-to-weeks) periods of direct sun can induce drought stress, and epiphyte species that live in this part of the Andes may possess adaptations for surviving drought, similar to those in lowland dry or seasonal forests ^36, 37, 68^. Epiphyte drought tolerance is higher in areas where drought occurs more frequently ^19, 69^, and many epiphyte species have adaptations for surviving drought – crassulacean acid metabolism, desiccation tolerance, pseudobulbs, succulent leaves and other water-storing organs. Consistent with this idea, *Elaphoglossum* ramets transplanted from 1500 m had higher survival than those from higher elevations, regardless of the transplant elevation ( Figure 2). The stronger response of epiphyte mats transplanted from normally cloud immersed elevations (i.e. 1800 m) compared to those transplanted from lower elevations suggests that lower elevation populations may be better adapted to drought stress due to less frequent cloud immersion. Another example of locally adapted epiphytes was observed in subtropical China, where bryophytes transplanted downslope lost biomass, while *in situ* measurements showed no change in biomass across the gradient ^38^.

## Conclusion

Greater epiphyte resistance to drought in this part of the Andes compared to previous studies may indicate that even seemingly benign dry seasons or dry periods can be important for structuring epiphyte communities, with potential implications for larger scale patterns of diversity. More generally, while epiphyte response to global climate change on tropical mountains is discussed in the literature ^18, 19, 29, 38^, tropical mountains and their climates are highly heterogeneous, and predictions may defy all but the broadest generalizations. Fundamental differences in the climate and biogeographical contexts may lead to differences in species response to climate change. Long-term experimental studies in tropical montane systems are needed to understand the drivers of patterns of epiphyte abundance, in particular why there is a change in biomass and abundance at putative ‘cloud base’ (which is correlated with changes throughout the ecosystem), and how these diverse communities will respond to climate change. While our experiment suggests that epiphytes in our study system show some resistance to climate change, climate models predict more severe droughts in parts of the Andes ^20, 21^. Pervasive changes in the tree canopy of the western Amazon following the 2005 Amazon drought persisted until an even stronger drought in 2010 ^70^; it is unknown whether similarly long-lasting effects were present in Andean cloud forest. Given the keystone position of epiphytes in cloud forests, drought-induced changes in epiphyte communities could have cascading effects throughout the ecosystem.

## Figures and Tables

**Figure S2.**
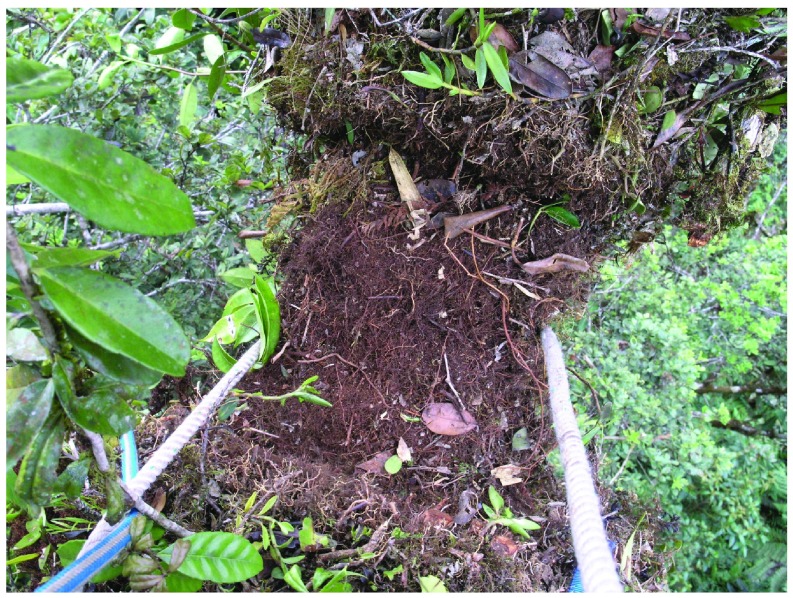
Mat removed. A transplanted mat was removed from this branch at 1650 m.

**Figure S3.**
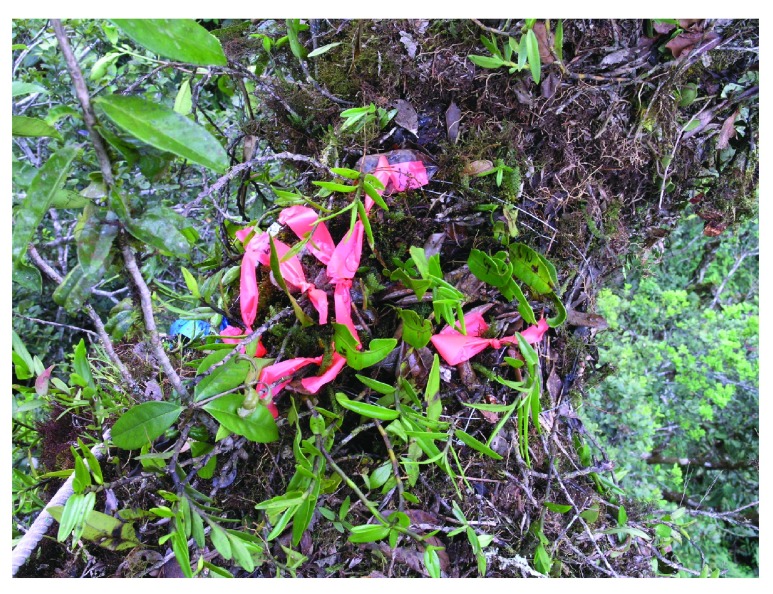
Transplanted mat. A mat from another tree was transplanted into the same location.
