# Supplementary material for: Epiphyte response to drought and experimental warming in an Andean cloud forest
Source: F1000Res. 2014 Jun 6;3:7. Originally published 2014 Jan 13. [Version 2] doi: 10.12688/f1000research.3-7.v2 (PMC4133766; doi:10.12688/f1000research.3-7.v2)
Supplement: Peer review history

# Peer review: Referee response for version 2

**DOI:** 10.5256/f1000research.4658.r5038

**Published:** 2014-07-21

**Authors:** Liang Song

**Affiliations:** 1 Xishuangbanna Tropical Botanical Garden, Chinese Academy of Sciences, Kunming, China

The article ‘Epiphyte response to drought and experimental warming in an Andean cloud forest’ has been strongly improved in both form and content since the previous version. I still have some minor suggestions for improvements.

**Introduction**:

- Paragraph 1: To be simpler and clearer, I think biodiversity hotspots is better than ‘hotspots of biological diversity’.
- Paragraph 4: In your abstract, you mentioned *‘Effects differed among species, but effects were generally stronger and more negative for epiphytes in mats transplanted downslope from the highest elevation, into warmer and drier conditions, than for epiphyte mats transplanted from other elevations. In contrast, epiphytes from lower elevations showed greater resistance to drought in all treatments*.’ as one of your main results. So, I think ‘Do epiphytes growing at lower elevations (drier, warmer condition) have greater drought tolerance than epiphytes growing at higher elevations’ is among the most important question addressed in your study, which should be highlighted in your aims.

 

**Discussion:**

- Paragraph 2: I do not totally agree that ‘Stronger effects would be expected for an experiment carried out over multiple years, since plants often react to stressful conditions through physiological responses such as closing stomata, which lowers carbon acquisition.’ because plants may also acclimate to new habitat over multiple years.

I have read this submission. I believe that I have an appropriate level of expertise to confirm that it is of an acceptable scientific standard.

# Peer review: Referee response for version 1

**DOI:** 10.5256/f1000research.3490.r3105

**Published:** 2014-03-11

**Authors:** Liang Song

**Affiliations:** 1 Xishuangbanna Tropical Botanical Garden, Chinese Academy of Sciences, Kunming, China

The purpose of this manuscript is to determine the potential impacts of drought and experimental warming on the performance of vascular epiphytes in a tropical montane cloud forest. There is a lack of information about the effects of climate change on epiphytes in mountain environments. The use of empirical rather than observational data as well as exchanging space for time to demonstrate those effects is also very commendable since such data is extremely rare.

Most studies of the effects of a changing climate on plants in mountain environments focus on changes to the tree line or tree species. However, the choice of epiphytes as experimental subjects in this study is also important, since in my opinion, they would be one of the first groups of organisms to reflect the effects of climate change. Epiphytes have a more direct link to atmospheric conditions and changes to those conditions than plants that root in the soil, and thus are more sensitive to moisture levels as noted by the authors.

This study clearly creates unique data on the impact of climate change on epiphytes and demonstrates an alternative, highly sensitive method of detecting changes in atmospheric conditions in mountain environments. The article is well constructed and clear and the statistical analyses appropriate. Considering that this experiment was conducted in the canopy, the authors must have made a great effort to finish it.

Although the underpinnings of this work are scientifically sound, and some results correlate well with data previously reported for vascular epiphytes by other authors ( Nadkarni and Solano, 2002), there are several major problems that I feel should be addressed:

1. The time span of the experiment is only one year, which is too short.
2. Microclimatic data was not monitored in the three studied elevations during the experimental period - which may obscure the interpretation of the results.
3. There should be more species with different functional types, which could support the third hypothesis that different functional types (e.g. strap-leaf ferns, orchids, ericaceous shrubs) do not respond similarly to moisture gradients. If it is not possible for you to find enough species I would suggest not using ‘functional types’.

 

I have provided a more detailed critique below:

**Title:**

- The title is well chosen although I would suggest adding the location where the study was conducted (e.g. “in the Andes cloud forest”). As noted by the authors, “ *Cloud forests vary worldwide, with differences in cloud base height and the proportion of moisture received by the vegetation via cloud stripping versus rainfall*” and “ *High rainfall in this part of the Andes may mean that epiphytes here are less dependent on cloud immersion to maintain their water balance than their counterparts in other cloud forests.*” -  This is not a general study concerned about all epiphytes, but a local study restricted to four typical vascular epiphytes in the Andes cloud forest.

**Abstract:**

- The abstract is basically well written, but the summary of the main findings do not seem appropriate. In the paper, the authors summarize that “ *Ramet mortality increased, recruitment decreased, and population size declined for epiphytes in mats transplanted down slope from the highest elevation, into warmer and drier conditions*”, which is not strongly supported by the results. In the results, the authors mention that “ *Across all species, there were no significant effects on survival of any of the treatments for mats transplanted across elevations (Table 5 and Figure 2). For recruitment and population change, there was a significant interaction between source and transplant elevation (Table 5), with both positively associated with elevation for mats transplanted from 1500 and 1800 m, but negatively associated with altitude for mats from 1650 m (Figure 2).*” Different epiphyte species and different parameters showed different response to treatments and sources. The results are much more complicated than what you summarized.
- The authors mentioned that their reciprocal transplant design allowed them to distinguish between the effects of moving mats away from their home elevation versus moving plants into lower moisture conditions. As a key control, results concerned about the transplant effect should be included in the abstract.
- The authors mention biomass loss in the last sentence, but this conclusion was based on other studies (I guess it is based on Song’s results), not their own.

 

**Introduction:**

- Paragraph 1: I suggest adding a sentence to illuminate why epiphytes are sensitive to water availability and temperature.
- Paragraph 4: The authors mention “ *Do epiphyte species of different functional types (e.g. strap-leaf ferns, orchids, ericaceous shrubs) respond similarly to moisture gradients*?” as one of the objectives of the study. If so, why were only four species studied? There is only one fern, one shrub, and two orchids in the experiment. It is not possible, in my opinion, to draw any useful conclusions of functional types with such a limited selection.
- Paragraph 5: this paragraph is repeating much of the fourth paragraph, I suggest combining the two.

 

**Materials and Methods:**

- The authors could provide a more detailed description of the four target genera, e.g., “What are the elevational ranges of the target genera in the Andes?” “What is the abundance of the four target genera at different elevations (1500m, 1650m, 1800m)?” etc. This information would be valuable to the interpretation of the results.
- Have the authors monitored the microclimatic data of the studied elevations during the experimental period? The details of this data are critical to the interpretation of the results yet it has not been included. Although the July climate for weather stations (Table 1) showed the general pattern of temperature and moisture level along the experimental altitudes it is not the microclimatic data of the study sites, especially as no data was provided at an elevation of 1650m.
- The time span of the experiment is only one year. Longer term monitoring (at least 2 years) at regular intervals (e.g. one year) would be much more valuable. In addition, I would suggest that the authors monitor other physiological parameters such as photosynthesis and chlorophyll fluorescence in their future work. Their conclusions would be much stronger based on these data.

 

**Discussion:**

- The title of the paper is “ *Epiphyte response to drought and experimental warming*”; however, the authors do not mention warming in the discussion section. The authors should focus the discussion section more on how different epiphytes respond to drought and warming in this study rather than the reasons for the relative resistance of epiphytes to moisture stress.
- The authors overstate their results in some places. For example, they mentioned “ *Vascular epiphytes transplanted down slope from our highest elevation had lower demographic performance when transplanted to the lowest elevation*” in the first sentence of the discussion section. This is only partly true for population change, while not true for survival and recruitment (Fig. 2). Although functional types are mentioned, the authors only have 4 species belonging to 3 functional types.
- Paragraph 1: “ *In general, it appears that epiphytes responded to water stress but we also found evidence for local adaptation.*” Both temperature and water availability changes along the elevations. Why are you sure that epiphytes responded to water stress not temperature?
- Paragraph 2 and 3: Rainfall compensation and drought tolerance seems self-contradictory.
- PET: You should provide the full name when it appeared for the first time.

I have read this submission. I believe that I have an appropriate level of expertise to confirm that it is of an acceptable scientific standard, however I have significant reservations, as outlined above.

# Response

**Published:** 2014-05-23

**Authors:** Joshua Rapp

**Affiliations:** Harvard University, USA

We thank Dr. Song for the reviewing our paper and providing constructive feedback. Below we respond to these comments and describe the changes made to the paper in response. Dr. Song’s comments are in [brackets].

1. [The time span of the experiment is only one year, which is too short.]
  While we agree that it would have been ideal to extend the study another year, this was not possible due to logistical constraints. Since the experiment was conducted in 2005-2006, it is not possible to add more time to the experiment. We now discuss the effect of experiment duration in the second paragraph of the Discussion.
2. [Microclimatic data was not monitored in the three studied elevations during the experimental period - which may obscure the interpretation of the results.]
  We agree that microclimate data during the experiment would have been ideal. Sensors placed at the study site during the experiment stopped recording data soon after deployment. We therefore used data from another period. We added a sentence to the Discussion noting that microclimate data was not available during the period, but refer to rainfall data from the nearby Rocotal climate station.
3. [There should be more species with different functional types, which could support the third hypothesis that different functional types (e.g. strap-leaf ferns, orchids, ericaceous shrubs) do not respond similarly to moisture gradients. If it is not possible for you to find enough species I would suggest not using ‘functional types’.]
  Since the experimental design focused on plots rather than individual species, we did not have control over the sample size for individual species. In this analysis we used the most abundant species in the plots, to maximize the statistical power to detect differences among treatments. We agree that this experimental design does not allow for a robust test of the hypothesis that functional types may differ in their response to moisture gradients. We therefore removed this hypothesis from the Introduction. However, we include the point in the Discussion, since the experiment does shed some light on differences among functional types, and we feel the patterns are worth mentioning. 

**Title:**

- [The title is well chosen although I would suggest adding the location where the study was conducted (e.g. “in the Andes cloud forest”). As noted by the authors, “ *Cloud forests vary worldwide, with differences in cloud base height and the proportion of moisture received by the vegetation via cloud stripping versus rainfall*” and “ *High rainfall in this part of the Andes may mean that epiphytes here are less dependent on cloud immersion to maintain their water balance than their counterparts in other cloud forests.*” -  This is not a general study concerned about all epiphytes, but a local study restricted to four typical vascular epiphytes in the Andes cloud forest.]

Title changed to “Epiphyte response to drought and experimental warming in an Andean cloud forest”

**Abstract:**

- [The abstract is basically well written, but the summary of the main findings do not seem appropriate. In the paper, the authors summarize that “ *Ramet mortality increased, recruitment decreased, and population size declined for epiphytes in mats transplanted down slope from the highest elevation, into warmer and drier conditions*”, which is not strongly supported by the results. In the results, the authors mention that “ *Across all species, there were no significant effects on survival of any of the treatments for mats transplanted across elevations (Table 5 and Figure 2). For recruitment and population change, there was a significant interaction between source and transplant elevation (Table 5), with both positively associated with elevation for mats transplanted from 1500 and 1800 m, but negatively associated with altitude for mats from 1650 m (Figure 2).*” Different epiphyte species and different parameters showed different response to treatments and sources. The results are much more complicated than what you summarized.]

We’ve changed to state that epiphytes moved down slope from the highest elevation had stronger and more negative response than epiphytes moved from other elevations. This also addresses the next comment.

- [The authors mentioned that their reciprocal transplant design allowed them to distinguish between the effects of moving mats away from their home elevation versus moving plants into lower moisture conditions. As a key control, results concerned about the transplant effect should be included in the abstract.
- The authors mention biomass loss in the last sentence, but this conclusion was based on other studies (I guess it is based on Song’s results), not their own.]

We revised the last sentence to focus on how the environmental and evolutionary context may affect epiphyte response to climate change.

**Introduction:**

- [Paragraph 1: I suggest adding a sentence to illuminate why epiphytes are sensitive to water availability and temperature.]

We added a clause to the sentence: “Cloud immersion is important for many epiphyte species to maintain a positive water balance and avoid desiccation” making it clear that: “this makes them sensitive to changes in moisture regimes.” In the next paragraph we describe how cloud formation and temperature are linked, and do not feel that it would add any clarity to the manuscript to repeat that here.

- [Paragraph 4: The authors mention “ *Do epiphyte species of different functional types (e.g. strap-leaf ferns, orchids, ericaceous shrubs) respond similarly to moisture gradients*?” as one of the objectives of the study. If so, why were only four species studied? There is only one fern, one shrub, and two orchids in the experiment. It is not possible, in my opinion, to draw any useful conclusions of functional types with such a limited selection.]

We deleted Question (3).

- [Paragraph 5: this paragraph is repeating much of the fourth paragraph, I suggest combining the two.]

We deleted the last two sentences referring to functional types. The rest of the paragraph elaborates on the approach outlined in the preceding paragraph. We feel that for clarity it is useful to first describe the research questions generally, and then operationalize the general questions. For this reason, we first describe how we expect performance to change with treatments, and then, in the next paragraph, describe the specific performance metrics used, and more specific predictions of how we expected them to change.  We have therefore left the structure as is, with one general and one more specific paragraph.

**Materials and Methods:**

- [The authors could provide a more detailed description of the four target genera, e.g., “What are the elevational ranges of the target genera in the Andes?” “What is the abundance of the four target genera at different elevations (1500m, 1650m, 1800m)?” etc. This information would be valuable to the interpretation of the results.]

All of the genera have broad ranges in Peru, encompassing the study elevations. However, this information is less useful than the elevational distributions of the morpho-species sampled. We have added text in a new section (Focal species) of the Methods to point out more clearly the differences in abundance with elevation of the different target taxa. Table 2 gives the abundance of each the target morpho-species at each elevation.

- [Have the authors monitored the microclimatic data of the studied elevations during the experimental period? The details of this data are critical to the interpretation of the results yet it has not been included. Although the July climate for weather stations (Table 1) showed the general pattern of temperature and moisture level along the experimental altitudes it is not the microclimatic data of the study sites, especially as no data was provided at an elevation of 1650m.]

Unfortunately, temperature and humidity sensors at the study site during the experiment stopped recording data soon after deployment, but this was not discovered until the end of the experiment. Therefore, microclimate data during the experiment is not available. Here we are reporting data for sensors deployed for a different study begun after the experiment that is the focus of this paper. We think this data is still useful, given that it is close to the experimental locations, and better than not reporting any climate data.

- [The time span of the experiment is only one year. Longer term monitoring (at least 2 years) at regular intervals (e.g. one year) would be much more valuable. In addition, I would suggest that the authors monitor other physiological parameters such as photosynthesis and chlorophyll fluorescence in their future work. Their conclusions would be much stronger based on these data.]

 While we agree that longer term monitoring would be ideal, this was not possible given logistical constraints. Here we are reporting what we did. We have added a sentence to the Discussion (2 ^nd^ paragraph) stating that physiological measurements would be helpful.

**Discussion:**

- [The title of the paper is “ *Epiphyte response to drought and experimental warming*”; however, the authors do not mention warming in the discussion section. The authors should focus the discussion section more on how different epiphytes respond to drought and warming in this study rather than the reasons for the relative resistance of epiphytes to moisture stress.]

We now mention temperature and moisture together in the first part of the Discussion, since it is not possible from our experimental design to separate the effects of each.

- [The authors overstate their results in some places. For example, they mentioned “ *Vascular epiphytes transplanted down slope from our highest elevation had lower demographic performance when transplanted to the lowest elevation*” in the first sentence of the discussion section. This is only partly true for population change, while not true for survival and recruitment (Fig. 2). Although functional types are mentioned, the authors only have 4 species belonging to 3 functional types. ]

This is a good point. We have changed the text to place less emphasis on functional types. We still mention differences in functional types as a potential factor in the species-level differences observed, but also include the caveat that our experimental design does not allow the rigorous testing of this hypothesis.

- [Paragraph 1: “ *In general, it appears that epiphytes responded to water stress but we also found evidence for local adaptation.*” Both temperature and water availability changes along the elevations. Why are you sure that epiphytes responded to water stress not temperature?]

Good point. See above.

- [Paragraph 2 and 3: Rainfall compensation and drought tolerance seems self-contradictory.]

These two effects do act in opposite directions, but both are possible, and non-mutually exclusive. Rainfall compensation could be the usual rule, with occasional drought also providing a selective pressure. We now describe this more clearly at the end of section 1 of the Discussion.

- [PET: You should provide the full name when it appeared for the first time.]

Thank you for catching this. Changed to potential evapotranspiration. We also deleted PET, since it only occurs once.

# Peer review: Referee response for version 1

**DOI:** 10.5256/f1000research.3490.r3588

**Published:** 2014-03-10

**Authors:** Peter Hietz

**Affiliations:** 1 Department of Integrative Biology, University of Natural Resources and Applied Life Sciences, Vienna, Austria

This manuscript addresses the question how epiphyte communities in tropical montane forests may be affected by climate change by transplanting epiphyte mats between forests at different altitudes. This is a reasonable proxy of what might happen under climate change with moderate temperature changes and substantial changes in cloud base height and thus available humidity.

The paper is well written, the experiment is clearly described and the analysis is logical (if complex).

I do suggest some additional thoughts and caveats - for the discussion mainly.

 

1. As one might expect, the outcome of such a study (four genera, three altitudes, three parameters evaluated) is complex, with significant and (mostly) non-significant effects. Overall the effect was perhaps less than expected (at least compared to a similar study by Nadkarni & Solano (2009) from Costa Rica). In this case, one tends to pick the significant effects that confirm expectations and ignore those that do not. The authors should avoid simplifying too much, particularly in the abstract, which is what most people will read. The abstract states that *"Ramet mortality increased, recruitment decreased, and population size declined for epiphytes in mats transplanted down slope from the highest elevation".* This appears to contrast with the results *("Across all species, there were no significant effects on survival of any of the treatments for mats transplanted across elevations"*). 
2. Details of the experimental species used are covered in the methods and results sections, however it seems that the plants were either identified only to the genus level or several species (identified or not) were included within each genus. Unless the authors are sure that only one species per genus was used, they should refer to the groups as "genus", and not "species". It is a substantial difference if we compare one species with individuals adapted to different climates within their phenotypic plasticity, or species differing in their evolutionary adaptations. This should be made clear in the methods section and acknowledged in the discussion. 
3. Under drought, plants or ramets may completely die off, but drought first results in weaker plants that exhaust their resources. As such, the effect of being transplanted to a different climate may take more than one year to produce significant effects on population size or also on other parameters studied. This should be acknowledged. 
4. The discussion suggests that epiphytes may be fairly drought tolerant in the forest studied because they must have survived unusually dry years (such as 2005, at the beginning of the experiment). This is a good point, and to put this into context it would be good to show precipitation trends (perhaps monthly sums for the closest station available) over several years preceding and including the experiment. 

**Minor comments:**

- Please explain how the daily photographs used to compare the cloud base height were taken and analyzed.
- The correct name is *Scaphyglottis*, not *Schaphyglottis*.
- Why does Table 4 have three elevations for *Elaphoglossum*, one for *Maxillaria* (all not significant) and none for the other two genera?”

I have read this submission. I believe that I have an appropriate level of expertise to confirm that it is of an acceptable scientific standard.

# Response

**Published:** 2014-05-23

**Authors:** Joshua Rapp

**Affiliations:** Harvard University, USA

We thank Dr. Hietz for taking time to review our paper and for the constructive comments. These have improved the paper. We respond to these comments below and describe the changes made to the paper in response to these comments. Dr. Hietz’ original comments are in [brackets].

1. [As one might expect, the outcome of such a study (four genera, three altitudes, three parameters evaluated) is complex, with significant and (mostly) non-significant effects. Overall the effect was perhaps less than expected (at least compared to a similar study by Nadkarni & Solano (2009) from Costa Rica). In this case, one tends to pick the significant effects that confirm expectations and ignore those that do not. The authors should avoid simplifying too much, particularly in the abstract, which is what most people will read. The abstract states that *"Ramet mortality increased, recruitment decreased, and population size declined for epiphytes in mats transplanted down slope from the highest elevation".* This appears to contrast with the results *("Across all species, there were no significant effects on survival of any of the treatments for mats transplanted across elevations"*). ]
  We have revised the text to be more precise regarding the observed results, especially in the Discussion. In the Abstract we now state that effects were stronger and more negative for epiphytes moved from the highest elevation as compared to those transplanted from other elevations. While this is certainly still a simplification of the results, it does accurately represent the results since significant effects were largely confined to mats transplanted down-slope.
2. [Details of the experimental species used are covered in the methods and results sections, however it seems that the plants were either identified only to the genus level or several species (identified or not) were included within each genus. Unless the authors are sure that only one species per genus was used, they should refer to the groups as "genus", and not "species". It is a substantial difference if we compare one species with individuals adapted to different climates within their phenotypic plasticity, or species differing in their evolutionary adaptations. This should be made clear in the methods section and acknowledged in the discussion. ]
  We identified each taxa to morpho-species. This is stated in the first paragraph of the *Data collection* section of the methods, but was obviously not clear enough. We used species after this point for brevity, but to make it clear to the reader, we have now changed ‘species’ to ‘morpho-species’ whenever referring directly to the taxa in the study. We have left ‘species’ in place where it is used in a more general discussion (i.e. when discussing species composition, species ranges, etc.). We were unable to identify taxa to species definitively because most species were not reproductive during the survey periods. Therefore, while we are fairly certain that our morpho-species were single biological species, it is possible that one or more contain multiple cryptic species.  We describe in more detail in the *Data collection* section why morpho-species were used, and add a sentence acknowledging that taxonomic uncertainty could have affected our results.
3. [Under drought, plants or ramets may completely die off, but drought first results in weaker plants that exhaust their resources. As such, the effect of being transplanted to a different climate may take more than one year to produce significant effects on population size or also on other parameters studied. This should be acknowledged. ]
  Very good point. We added a discussion of this idea to the second paragraph of the Discussion (this is a new paragraph).
4. [The discussion suggests that epiphytes may be fairly drought tolerant in the forest studied because they must have survived unusually dry years (such as 2005, at the beginning of the experiment). This is a good point, and to put this into context it would be good to show precipitation trends (perhaps monthly sums for the closest station available) over several years preceding and including the experiment.] 
  Added figure (Figure 4) of monthly rainfall at the closest climate station (Rocotal, 2010m elevation) to the Discussion.

**Minor comments:**

- [Please explain how the daily photographs used to compare the cloud base height were taken and analyzed.]

Now explained in the Methods.

- [The correct name is *Scaphyglottis*, not *Schaphyglottis*.]

Thank you. Fixed throughout.

- [Why does Table 4 have three elevations for *Elaphoglossum*, one for *Maxillaria* (all not significant) and none for the other two genera?”]

Table 2 shows the abundance of each morpho-species in the initial plots at each elevation. For the ‘within-species’ analysis, we only included source elevations where the morpho-species was abundant (bold in Table 2). Our criteria for “abundant species” is now given in the Methods.
